# Recent Advances in Searching for DNMT Inhibitors and Their Potential Application in Treating Human Diseases

**DOI:** 10.3390/ijms27104560

**Published:** 2026-05-19

**Authors:** Anatoliy A. Bulygin, Anastasiia T. Davletgildeeva, Nikita A. Kuznetsov

**Affiliations:** 1 Institute of Chemical Biology and Fundamental Medicine, Siberian Branch of the Russian Academy of Sciences, 630090 Novosibirsk, Russia; abulygin@1bio.ru (A.A.B.); a.davletgildeeva@1bio.ru (A.T.D.); 2 Department of Natural Sciences, Novosibirsk State University, 630090 Novosibirsk, Russia

**Keywords:** DNA methyltransferases, DNA methylation, inhibitors, drug design, CpG dinucleotides

## Abstract

DNA methylation is one of the most important epigenetic mechanisms regulating gene expression. DNA methyltransferases (DNMTs) are key players in these processes, regulating dynamic DNA methylation patterns in embryonic and adult cells. Therefore, dysfunction of DNMTs can lead to serious diseases and cancer due to distortions in gene methylation, including that of tumor suppressor genes. Due to the reversibility of DNA methylation, DNMTs are considered an important epigenetic target for drug development. This narrative review summarizes knowledge about DNMTs, including structural features and biological functions, and describes the development of DNMT inhibitors as therapeutics, from the earliest developments to the most modern and promising ones.

## 1. Introduction

One of the most studied and most important epigenetic marks that regulate gene expression is symmetric C5 cytosine methylation in DNA CpG-dinucleotides [1,2]. Despite the fact that this mark is widespread in many eukaryotic organisms, its levels vary greatly among different species [3]. C5 methylation of CpG dinucleotides’ cytosine plays an extremely important role in the regulation of gene product expression, chromatin structure and many other biologically relevant processes, such as X-chromosome inactivation and retrotransposon silencing [4]. Epigenetic methylation of cytosine is catalyzed by the family of DNA methyltransferases (DNMTs). To establish the initial pattern of DNA methylation, the de novo DNA methyltransferases DNMT3A and DNMT3B are involved [5,6,7,8,9]. After the proper DNA methylation pattern has been set it is then maintained by DNMT1, which is responsible for the methylation of the newly synthetized DNA chain [8]. Undesirable changes in the DNA methylation pattern, including those due to dysregulation of the activity of enzymes that establish and maintain it, are currently associated with the development of many human diseases, including a number of severe developmental disorders and cancer [10]. This makes the enzymes participating in epigenetic DNA methylation potent targets for developing the inhibitors.

The main problem with currently available DMNT inhibitors is their low selectivity, which means that their use carries a high risk of unnecessary side effects. Therefore, understanding the subtle mechanisms that influence the methyltransferase activity of DMNTs, their specificity for certain genomic loci, and the interaction of these enzymes with partner proteins is necessary for the development of a new generation of precise and effective DMNT inhibitors.

The objective of this narrative review is to summarize knowledge about DNMTs, including their structural features and biological functions, and describe the development of DNMT inhibitors as therapeutics, from the earliest developments to the most modern and promising ones. We used the PubMed database (https://pubmed.ncbi.nlm.nih.gov/) as the main source of information with keywords: “DNMT”, “human disease”, “structure”, “nucleoside”, “repurposed”, “natural”, “DNA binding”, “SAM binding”, “active site binding”, “allosteric”, and “inhibitor”. The selection of articles includes all of the relevant articles published between 1975 and 2025.

## 2. Domain Organization of DNA Methyltransferases

**Figure 1:**
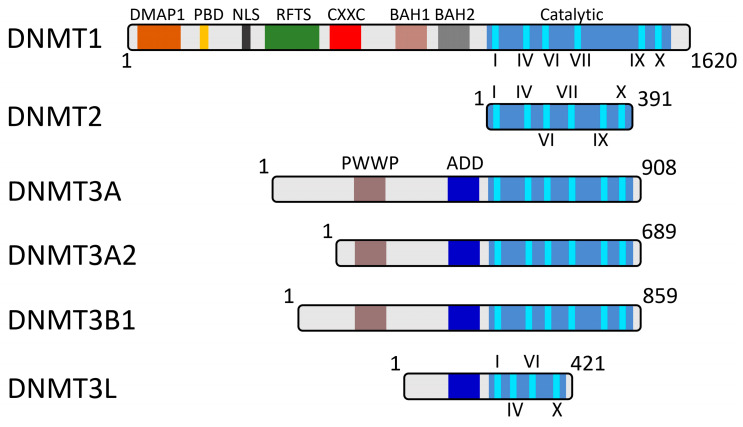
Schematic representation of the conserved domains in human DNMT enzymes. The N-terminal domain of DNMT1 includes seven important sequences: DNA methyltransferase-associated protein-1 binding domain (DMAP1), proliferating cell nuclear antigen (PCNA) binding domain (PBD), nuclear localization signal (NLS), replication foci-targeting sequence (RFTS) domain, cysteine-rich subdomain (CXXC) and two bromo-adjacent-homology (BAH) domains. DNMT3 enzymes possess Pro-Trp-Trp-Pro (PWWP) and ATRX-DNMT3-DNMT3L (ADD) domains, except DNMT3L, which only has an ADD domain. The C-terminal domain of all of the DNMTs is composed of the subdomains required for their catalytic activity. These sequences, numbered from I to X, are required for SAM binding (I, II, and III), cytosine binding and catalysis (IV and VII) and cofactor binding (I, X) [15]. The C-terminal domain of DNMT1 and DNMT3A/3B contains gene sequences I through X, while DNMT3L contains only gene sequences I, IV, VI, and X.

There are five main DNA methyltransferases found in mammalian cells (Figure 1). Three of them, the maintenance methyltransferase DNMT1 and the de novo methyltransferases DNMT3A/3B, possess DNA methyltransferase activity toward cytosine in the CpG dinucleotide context, although it was also shown that DNMT3A is able to perform non-CpG methylation too [4,11,12]. There are also DNMT2 and DNMT3L homologues which do not possess DNA methyltransferase activity, so they are considered noncanonical [7]. Nevertheless, it was shown by Goll et al. that DNMT2 actually is a tRNA methyltransferase, as it was able to methylate cytosine 38 in the anticodon loop of aspartic acid tRNA [13]. Except DNMT2, all of the DNMT homologues consist of a distinguishable regulatory N-terminal domain and catalytic C-terminal domain (Figure 1). The catalytic methyltransferase (MT) domain includes six sequence motifs (I, IV, VI, VIII, IX, and X), highly conserved among all DNMTs, separated by less conserved regions [14].

The enzymatic activity of DNMTs can be regulated through allosteric interactions between separate domains of the enzyme as well as through the interaction with other regulatory protein partners [15]. To provide methylation, DNMTs perform a catalytic transfer of a methyl group from a donor (S-adenosylmethionine (SAM)) to an acceptor C5 cytosine. The target cytosine is flipped out of the DNA double strand upon this process [16].

## 3. Role of the Main DNA Methyltransferases in Human Diseases

### 3.1. DNMT1’s Role in Human Organisms in Normal and Pathological States

Experiments in mice have clearly demonstrated that maintaining the epigenetic methylation levels above certain thresholds is vital for embryonic development, with DNMT1 deficiency causing early embryonic lethality and severe developmental defects [8,17,18]. DMNT1 is the only methyltransferase in mammals that is responsible for maintenance of DNA methylation levels established during early embryogenesis [7]. Indeed, it was established that different single nucleotide polymorphisms (SNPs) of the *DNMT1* gene are connected to the development of severe growth and developmental disorders, including disorders of the central and peripheral nervous systems, the consequences of which are observed immediately at birth, or arise and worsen over the course of a person’s life [19,20,21,22,23,24,25].

Inherited DNMT1 mutations in humans predominantly affect the regulatory RFTS domain and give rise to adult-onset neurodegenerative syndromes such as autosomal dominant cerebellar ataxia, deafness and narcolepsy (ADCA-DN) and hereditary sensory and autonomic neuropathy type 1E (HSAN1E) [18,21,24,26]. In these disorders, amino acid substitutions destabilize DNMT1 folding, alter the balance between auto-inhibition and DNA binding, reduce catalytic efficiency or, in some variants, enhance DNA affinity, ultimately disturbing maintenance methylation of DNA, most clearly having a negative effect on vulnerable neuronal populations [18,21,24,26,27,28,29]. The increased interaction of certain mutant forms with the E3 ubiquitin ligase UHRF1 and the tendency of mutant DNMT1 to aggregate likely further contribute to neurotoxicity and progressive neurological decline [18,28,29].

Beyond monogenic syndromes, DNMT1 dysregulation has been implicated in psychiatric and pain-related conditions. Conditional forebrain knockout of *DNMT1* in mice exerts anxiolytic and antidepressant-like effects, whereas pharmacological inhibition of DNMTs with nucleoside analogues such as 5-azacitidine and decitabine attenuates excessive alcohol consumption and neuropathic pain, suggesting that DNMT-dependent hypermethylation contributes to maladaptive plasticity in mood changes and addictive behavior in human [30,31] and model organisms [32,33]. Genetic association studies also indicate that polymorphisms in *DNMT1* and other *DNMT* genes may influence susceptibility to schizophrenia and Alzheimer’s disease, in line with elevated DNMT1 or DNMT3A mRNA levels detected in blood samples from affected patients [34,35,36,37].

Aberrant DNMT1-mediated methylation plays its destructive role in many other processes aside from its role in neurons and the nervous system. In heart failure, the development of fibrosis is accompanied by increased DNA methylation and the upregulation of DNMT1, DNMT3A and DNMT3B in cardiac fibroblasts under ischemic and hypoxic conditions, where HIF-1α-dependent DNMT induction promotes the expression of profibrotic genes such as α-smooth muscle actin and drives fibroblast activation and proliferation [38,39,40,41]. Elevated DNMT1 expression has also been reported in femoral tissues in disuse osteoporosis and in Graves’ disease, where overexpression may contribute to aberrant methylation of immunoregulatory genes and autoimmunity [42].

Apparently, one of the reasons for the observed association of methylation pattern disturbances with aging and the development of cancer is hypomethylation of CpG dinucleotides in regions of repetitive DNA sequences. One of the direct consequences of this process is the sleeping retrotransposon activation and disruption of the genomic stability of the cell [43]. Progressive DNA hypomethylation may be a consequence of decreased activity or amount of DNMT1 in cells over time [44]. Nevertheless, it was demonstrated that DNMT3A/3B, at least partially, are able to compensate for DNMT1’s function loss in cells [45,46,47]. On the other hand, elevated levels of DNMT1 were detected in metastatic breast cancer [48], as well as the number of SNPs associated with this oncological disease [49]. Gassenmaier et al. studied the DNMT1 expression in melanocytic tumors at different stages of proliferation and demonstrated, that the level of DNMT1 expression strongly correlates with proliferation in melanocytic tumors and increases with melanoma progression [50]. Overall, DNMT1 acts as a guardian of epigenetic stability in normal tissues, whereas its deregulation or mutation contributes to a spectrum of neurodegenerative, psychiatric, fibrotic and malignant diseases.

### 3.2. DNMT3A’s Role in Human Organisms in Normal and Pathological States

It was established that one of the specific functions of DNMT3A is the methylation of the *Xist* gene on the X chromosome and imprinted genes in germ cells [51]. Mice deficient in DNMT3A demonstrate normal development at early life stages but do not survive through the 4-week threshold [8]. Moreover, it was recently demonstrated that DNMT3A (specifically its DNMT3A2 isoform) plays an important role in long-term memory formation in mice [52].

As in the case of DNMT1, the important role of an adequate activity of DNMT3A in the nervous system is reflected in human overgrowth and neurodevelopmental disorders. Multiple DNMT3A SNPs affecting the PWWP, ADD and catalytic domains cause Tatton–Brown–Rahman syndrome, characterized by overgrowth and intellectual disability, with most substitutions reducing enzyme activity by disrupting interdomain communication or histone binding [53,54]. Many of these variants are also found in acute myeloid leukemia (AML), and some, such as the R771Q substitution, can increase DNMT3A activity several-fold compared to the wild-type enzyme, illustrating that both loss- and gain-of-function mutations contribute to leukemogenesis [53,55,56]. Furthermore, PWWP-domain substitutions W330R and D333N, found in microcephalic dwarfism patients, do not abolish the catalytic activity of the enzyme but disrupt its binding to histone H3 with di- and trimethylated Lys36 (H3K36me2/3). There is also an increase in the methylation of regions of the genome associated with Polycomb, particularly developmental genes [57].

Zhang et al. demonstrated the role of DNMT3A in the development of addictive-like behavior in rats [58]. It was shown that the expression of DNMT3A in the hippocampal CA1 region of rats was significantly upregulated after morphine self-administration. Moreover, microinjection of 5-aza into the hippocampal CA1 significantly attenuated the acquisition of morphine self-administration by the animals. Knockdown of DNMT3A also impaired the ability to acquire the morphine self-administration. Qian et al. also showed that use of 5-aza in combination with a reward memory retrieval approach after heroin administration resulted in reduced addictive behavior in rats [59]. These findings indicate that DNMT3A-dependent methylation in the limbic circuitry supports the consolidation of drug-associated memories.

Aside from AML, mutations in the *DNMT3A* gene are also often found in other hematological tumors, such as myelodysplastic syndrome and T-cell acute lymphoblastic leukemia [60,61]. Moreover, the most frequent substitutions tends to be of Arg882, which occurs in 30% of AML cases, and leads to a decrease in DNMT3A activity [62,63]. The resulting hypomethylation of CpG dinucleotides at certain loci leads to a disruption of the differentiation process of hematopoietic cells, which, apparently, initiates the process of malignant degeneration of blood cells [3]. In low-grade gliomas, one of the most frequently mutated enzymes participating in the regulation of DNA methylation/demethylation is DNMT3A, while DNMT3B and DNMT1 are mostly upregulated in such tumors [64]. Increased DNMT3A and DNMT3B expression has also been reported in breast cancers, primarily in early stages of tumorigenesis [48].

The functional consequences of DNMT3A dysregulation in cancer appear to be context-dependent. DNMT3A-driven tumorigenesis in breast cancer has been linked to hypermethylation of key loci, including *ERα* and *BRCA1* promoters [65], while overexpression of DNMT3A leads to suppression of the oncoprotein SOX2 expression, which in turn slows down the development of breast cancer cells [66]. This result is also supported by the fact that increased DNMT3A expression has been associated with better prognosis for patients with lung adenocarcinoma [67]. DNMT3A overexpression in synovial tissues has also been associated with hip osteoarthritis in rat models, suggesting a broader involvement of this enzyme in degenerative joint disease [68]. Collectively, these data support a dual role for DNMT3A as both an oncogenic driver and a context-dependent tumor suppressor, depending on the cellular environment and target loci.

### 3.3. DNMT3B’s Role in Human Organisms in Normal and Pathological States

It was shown in murine models that depletion of DNMT3B activity results in embryonic lethality combined with many growth and neurological defects [8]. These data, together with the data on changes in the methylation levels of specific gene regions in the absence of DNMT3B activity in mouse embryos, indicate that, for mammals, at least for mice, this enzyme plays a predominant role in de novo methylation during embryogenesis [69]. Herewith, double knockout of DNMT3A/3B leads to the development of even more defects compared to knockout of DNMT3B methyltransferase alone [8].

Mutations in *DNMT3B* are often found in patients with immunodeficiency, centromere instability, and facial abnormality (ICF) syndrome [8,70,71]. Moreover, all three currently known substitutions associated with the development of this syndrome are found in the MTase catalytic domain of DNMT3B.

In addition, some *DNMT3B* SNPs are associated with a higher risk of breast cancer development, but the variants registered to date are located at the promotor or intronic regions of the gene [72,73]. It is interesting that the amplification of the *DNMT3B* gene was found in breast cancer cells [74]. DNMT3B plays a protumorigenic role in human melanoma and loss of DNMT3B dramatically suppresses melanoma formation, as it was demonstrated in murine melanoma models [75]. It was shown that all three active DNMTs, DNMT1 and DNMT3A/3B, are overexpressed in colon tumor cells compared to normal tissues [76].

Yang et al. showed that DNMT3B participates in the epigenetic regulation of macrophage polarization, thus influencing their pro- or anti-inflammatory status in adipose tissue [77]. The authors found that DNMT3B expression was significantly elevated in pro-inflammatory M1 macrophages in obese subjects, whereas the knockdown of DNMT3B promoted macrophage polarization to an alternatively activated M2 phenotype and significantly improved adipocyte insulin signaling. It was also shown that the role of myeloid DNMT3B in macrophage polarization can influence the development of pulmonary fibrosis [78].

It is interesting that overexpression of all three DNA methyltransferases, DNMT1, DNMT3A and DNMT3B, in CD4+ T-cells was demonstrated to play an important role in the ovalbumin sensitization in the allergic rhinitis murine models [79].

Together, these findings highlight DNMT3B as a key de novo methyltransferase required for normal development, whose dysregulation contributes to immunodeficiency syndromes, cancer and inflammatory diseases.

### 3.4. DNMT2’s Role in Human Organisms in Normal and Pathological States

Until recently, DNMT2, a DNMT homologue consisting exclusively of the catalytic MT domain [14], was thought to possess no catalytic activity at all [7]. Nevertheless, the most recent data on this subject indicate that DNMT2 is an RNA methyltransferase rather than a DNA methyltransferase [13,80]. DNMT2 was shown to methylate cytosine 38 in the anticodon loop of aspartic acid tRNA. This modification is thought to take part in the immune response against RNA viral infections [81,82,83]. The role of DNMT2 in human diseases is yet to be established. However, this topic has recently begun to be addressed in the literature. Thus, DNMT2 was demonstrated to be up-regulated in low-grade glioma [64] and the knockout of *DNMT2* in glioblastoma cancer cells led to decreased levels of drug sensitivity [84].

A concise overview of the main physiological functions and major pathological associations of human DNMTs is provided in Table 1.

## 4. Structure of DNA Methyltransferases

### 4.1. Structure and Functions of DNMT1

**Figure 2:**
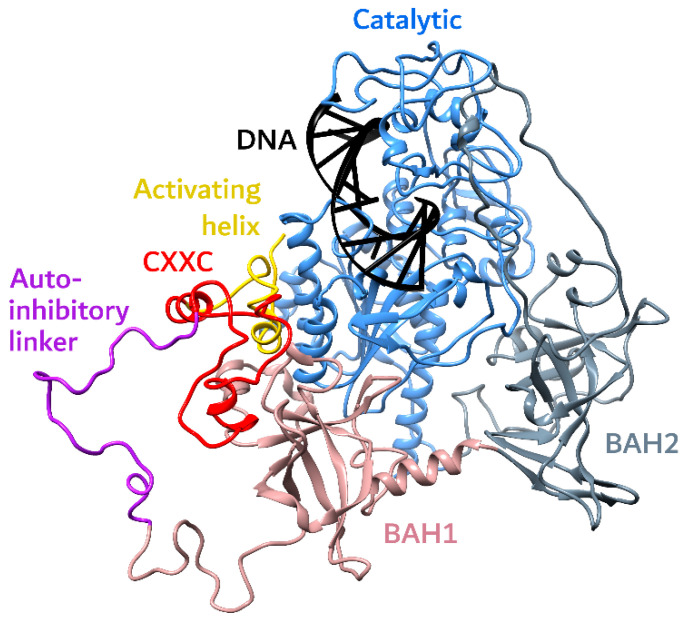
Structure of human DNMT1 (PDB ID 7XI9).

Human DNMT1 is quite a large protein of 1616 amino acid residues in total, and its domain structure, in general, includes the positively charged DMAP1, which is a component of the acetyltransferase complex, playing an important role in maintaining the function of embryonic stem cells, RFTS domain, CXXC, two BAH domains, and a C-terminal MT domain (Figure 2) [89]. PBD is also located at the N-terminal domain of DNMT1, and it ensures the interaction of the enzyme with the DNA replication machinery [3]. DNMT1 is highly conserved and is represented among all vertebrates [90]. One of the helixes in the catalytic C-terminal MT domain is distinguished as a DNA recognition helix. Between the CXXC subdomain and BAH1, there is a small auto-inhibitory linker which recognizes the DNA binding region and participates in the auto-inhibition of the DNMT1 along with the binding of the RFTS to the C-terminal MT domain [91,92]. Double monoubiquitylation at Lys18 and Lys23 on histone H3 (H3Ub2) by the E3 ubiquitin ligase (ubiquitin-like containing PHD and RING finger domains 1, UHRF1) rescues DNMT1 from this autoinhibition state [93,94,95,96]. Kikuchi et al. revealed details of this mechanism [89]. It was shown that in its core, there are simultaneous structural rearrangements affecting several inhibitory modules, namely RFTS, CXXC and auto-inhibitory linker. The authors suggested that H3Ub2 binding destabilizes the inhibitory interaction between the RFTS domain and the catalytic MT domain, which gives the possibility for hemimethylated substrate DNA to penetrate the catalytic core. A crucial role of the hydrophobic “Toggle” pocket in the stabilization of both the inhibited and the activated states of DNMT1 was also shown in that work. In the inhibited state, this hydrophobic pocket is bound to inhibitory phenylalanines in the DNA recognition helix. As for the switch to the activated state, “Toggle” accepts two phenylalanine residues from the activating helix, located between RFTS and CXXC, which was recently recognized as highly conserved [89]. At the same time, this helix is unique to DNMT1, making it a prospective target for novel inhibitor design.

Until recently, all resolved DNMT1 structures were obtained with proteins lacking the N-terminal region, specifically from amino acid 350 to the C-terminal domain [89,91,92,93,96,97,98,99,100,101,102]. Hu et al. reported the identification of the folded α-helical domain at the N-terminus of human DNMT1 by means of nuclear magnetic resonance (NMR) spectroscopy and small-angle X-ray scattering (SAXS) [90]. It is possible that this folded domain possesses a regulatory role. Thus, it was shown that the loss of this fragment in the DNMT of mammalian oocytes, due to the alternative RNA splicing of a sex-specific exon, leads to this protein being excluded from the cytoplasm [103]. Another example is that the deletion of this N-terminal fragment from DNMT1 leads to the diminishing of the inhibition of histone deacetylase-induced ubiquitylation-dependent degradation of DNMT1 and genomic hypermethylation as a consequence in breast cancer cell lines [104]. The most interesting fact is that the DNMT1 isoform that lacks the N-terminal domain possesses higher stability compared to the full-length DNMT1 in vivo [105,106]. The authors presenting the structure of the N-terminal domain suggested the facilitation of the possible regulatory functions of this fragment through protein–protein interactions [90].

The MT catalytic domain of DNMT1 plays an important role in the binding of the DNA substrate and a methyl group donor, SAM, and facilitates catalytic transfer of the methyl group to the targeted cytosine [107]. DNMT1 is a highly processive enzyme that can methylate up to three dozen CpG dinucleotides before dissociating from DNA [108,109]. This enzyme exhibits high specificity for methylation of cytosine at the fifth position in the context of hemimethylated CpG dinucleotides, as it is a maintenance methyltransferase. However, it has been shown that asymmetric methylation outside of CpG sites by this methyltransferase is also possible [110]. Thus, it is implied that the RFTS domain of DNMT1 may be responsible for targeting the enzyme on the hemimethylated sites [111]. At the same time, it is the CXXC domain which recognizes unmethylated CpG dinucleotides in DNA [3]. In addition to its key role in autoinhibition of DNMT1 and its substrate specificity, the RFTS domain is also known to be involved in DNMT1 dimerization and the localization of the enzyme at the heterochromatin region during the G2 phase [112].

The MT catalytic domain of all three catalytically active DNMTs contains 10 highly conserved motifs. The first three are involved in SAM binding, and considered motifs IV and V are absolutely necessary for catalysis [16,113]. The DNA recognition helix is located between the start of the VII motif and the IX motif’s end [114].

Despite the fact that both BAH domains of DNMT1 are highly conserved, the information available about them at the moment is very scarce [115]. However, the available data indicate that the interaction of the BAH domain with the repressive mark—trimethylated Lys20 of histone H4 (H4K20me3)—takes part in heterochromatin formation [99].

In addition, the structure of the catalytic domain contains a subdomain called “target recognition domain” (TRD). TRD takes part in DNA binding and contributes to the enzyme’s substrate specificity [116,117]. DNMT1 contains a large TRD—about 200 amino acid residues. Two protein loops are involved in the interaction with a semimethylated CpG—TRD loop I (residues 1501–1516) and TRD loop II (residues 1530–1537), which occupy a large region on the major groove side of the DNA. TRD loop I binds to the unmethylated DNA strand, while TRD loop II binds to 5-methylcytosine (m^5^C) and fully surrounds it.

### 4.2. Structure and Functions of DNMT3A/3B

**Figure 3:**
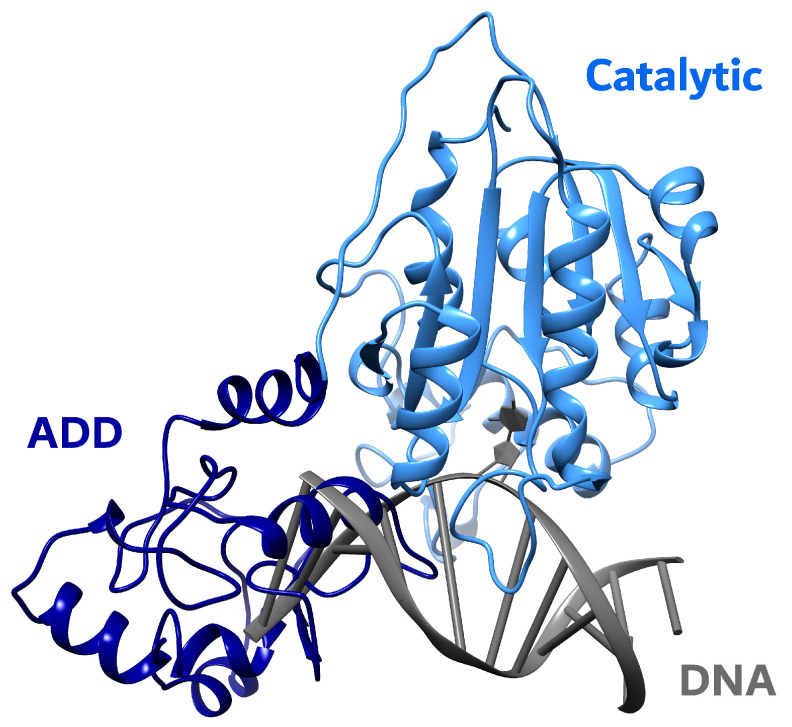
The structure of human DNMT3B (PDB ID 8EIH). The DNA molecule was added from the alignment with the structure of the DNMT1-DNA complex (PDB ID 7XI9) and has steric clashes with the ADD domain.

DNMT3A/3B possess a C-terminal catalytic region that is highly homologous to that of DNMT1, whereas the N-terminal domain that is also playing a regulatory role is quite distinct from the corresponding DNMT1’s domain. DNMT3A and DNMT3B are very close in amino acid sequence. The N-terminal domain of these enzymes includes the conservative PWWP and the ADD domains (Figure 3) [118]. The PWWP domain of DNMT3A and DNMT3B participates in the DNA binding, but it was shown that in the case of DNMT3A, the binding is one magnitude less effective [119,120]. It was shown that PWWP, precisely, is responsible for the heterochromatin localization of DNMT3A/3B through its binding to H3K36me2/3, and thus, for the DNA methylation by these enzymes [121,122]. It is interesting that the PWWP domain of DNMT3A was also shown to interact with thymine DNA glycosylase (TDG), which leads to the inhibition of the methyltransferase activity of DNMT3A [123]. Moreover, it was shown that another DNA glycosylase, methyl-CpG binding protein 4 (MBD4), cooperates with DNMT1, and they both are recruited at sites of oxidation-induced DNA damage and participate in controlling gene expression [124].

The ADD domain also anchors DNMT3A/3B to heterochromatin, but it interacts with the N-terminal region of H3 when its Lys4 is unmethylated [125]. It was shown that DNMT3A/3B enzymes are not selective and do methylate non-CpG dinucleotide sites in embryonic stem cells. Therefore, the destiny of the regions to be methylated (or not) is determined by factors such as the protection of CpG-rich regions and enhancers by H3K4 methylation and binding of certain transcription factors to DNA [7]. The ADD domain of DNMT3A was shown to bind many other proteins besides histone termini, including oncogene c-Myc [126], heterochromatin protein 1 beta (HP1β), DNA-binding transcriptional repressor protein RP58 and Lys9 of histone H3 (H3K9) methylase Suv39h1 [127].

The full length of human DNMT3A is 912 amino acid residues, but it is expressed in two isoforms, and the second one (DNMT3A2) is 223 residues shorter from the N-terminus. DNMT3A is expressed ubiquitously in organisms, but its levels are relatively low [128]. DNMT3A2 is mostly found in embryonic stem cells, early embryos, and developing germ cells in mice, and in human embryonal carcinoma cells. It is also detectable at some levels in the thymus and spleen [128].

DNMT3B is produced in multiple isoforms due to the alternative splicing of its gene, and not all of the isoforms are catalytically active [70,118,129,130,131]. DNMT3B1, the most active and the longest among the isoforms, consists of 853 amino acid residues in humans. It seems that catalytically inactive isoforms of DNMT3B nevertheless play regulatory roles in DNA methylation [3].

**Figure 4:**
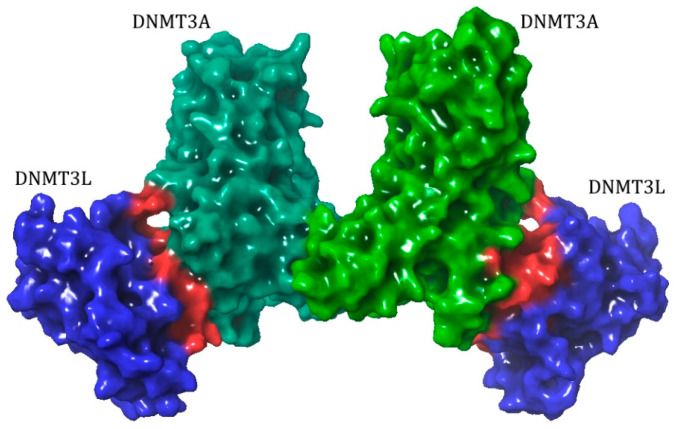
Structure of the DNMT3A-DNMT3L tetramer (PDB ID 4U7T). Proteins have large surface contact areas (highlighted in red) between their catalytic domains.

It was shown that the activation of the DNMT3A/3B enzymes is facilitated through the formation of a heterotetramer with a noncanonical DNMT3L catalytic-like domain (Figure 4) [85,86,87,88]. There are some similarities between the mechanism of the DNMT3A/3B activation by DNMT3L and the activation of the DNMT1 [89]. The thing is that the interactions between DNMT3s are formed through a series of hydrophobic contacts involving phenylalanine residues, which are known as the F-F interface [86]. The hydrophobic amino acid residues at the catalytic domain of DNMT3A/B and in the DNMT1 “Toggle” pocket are spatially corresponding. It is possible that this mechanism of the activation of DNMTs by covering the hydrophobic pocket of the catalytic domain could be an evolutionarily conserved solution for modulating the DNA methyltransferase activity [89].

It is also established that DNMT3A accommodates autoinhibitory conformation similarly to the DNMT1 enzyme. In this case, the binding of DNA at the catalytic site of DNMT3A is blocked by the interactions of the AAD and catalytic domains. The binding of the AAD to the H3 with unmethylated Lys4 leads to the initiation of a series of conformational changes within DNMT3A, which eventually results in its release from the autoinhibited state [132].

**Figure 5:**
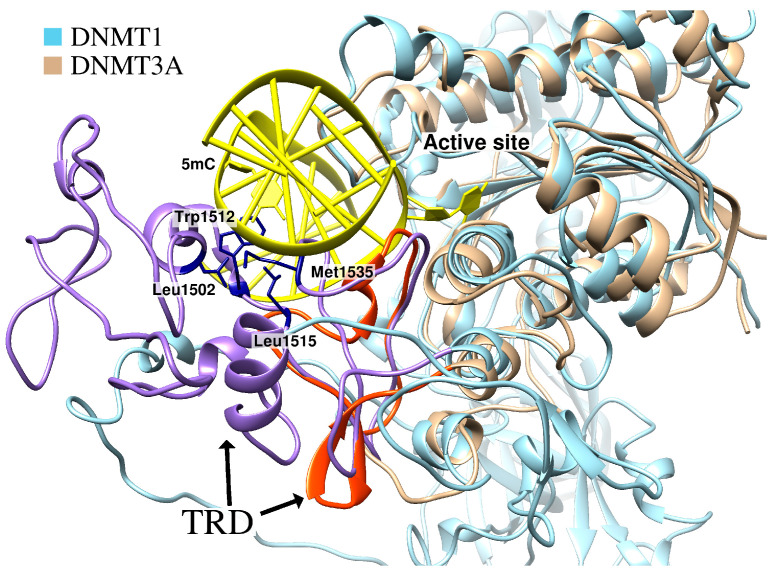
Comparison of the TRD structures of DNMT1 (cyan, PDB ID 4DA4) and DNMT3A (beige, PDB ID 6BRR). The TRD of DNMT1 (violet) is significantly larger than the TRD of DNMT3A (orange). Residues Leu1502, Trp1512, Leu1515 and Met1535 form a small but complete hydrophobic pocket around m^5^C.

Similarly to DNMT1, DNMT3A/3B also contain a TRD, but it is much smaller because there is only one protein loop in this domain that is analogous to TRD loop II. The second loop capable of interacting with m^5^C is missing, which may explain the difference in substrate specificity between these enzymes [133]. Differences in the structure of the TRD of DNMTs are the key features to consider during the design of specific inhibitors. The additional TRD loop of DNMT1 possesses three residues (Leu1502, Trp1512, Leu1515) that, together with Met1535 from another loop, form a complete hydrophobic pocket around the methylated cytosine on the major groove side of the DNA (Figure 5).

### 4.3. Structure of the Active Site of DNMTs and the Mechanism of Catalysis

**Figure 6:**
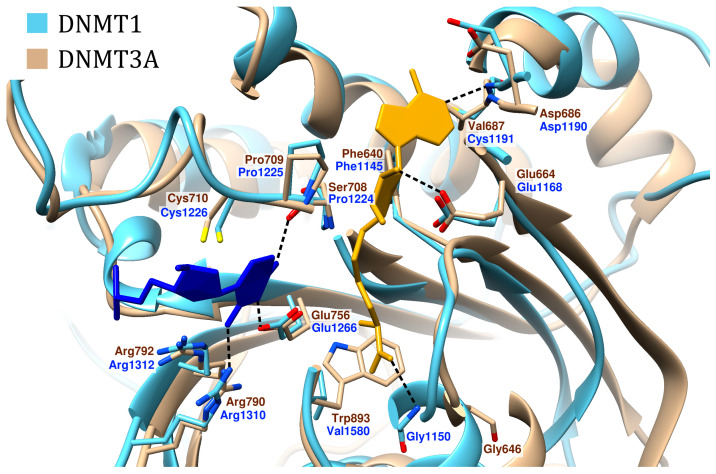
Comparison of the active site structures of DNMT1 (cyan, PDB ID 4DA4) and DNMT3A (beige, PDB ID 6BRR). The everted cytosine is shown in blue, SAM is shown in yellow, and H-bonds are represented as black dashed lines. This comparison also applies to the DNMT3B, which is very similar to DNMT3A.

At the active site of the enzyme, target cytosine forms hydrogen bonds (H-bonds) with the residues of glutamate, two arginines and also Ser708/Pro1224 in the case of DNMT3A and DNMT1, respectively (Figure 6). Moreover, catalytic residues Cys710/Cys1226 and Pro709/Pro1225 are also in close proximity to the base to be methylated. The H-bond between the cytosine and Ser708/Pro1224 is formed with the backbone oxygen atom of the residue instead of the side chain; thus, the immediate surroundings of the base may be considered fully similar for all three enzymes—DNMT1, DNMT3A and DNMT3B.

Due to its length, SAM occupies a large volume of the active site and interacts with two groups of residues. The aminoacidic part of SAM has contact with Glu756/Glu1266, Trp893/Val1580 and Gly646/Gly1150, and is capable of forming H-bonds with all three of them (Figure 6). The nucleoside part of SAM forms H-bonds with Glu664/Glu1168 and Val687/Cys1191, forms stacking interactions with Phe640/Phe1145, and is located close to Asp686/Asp1190.

**Figure 7:**
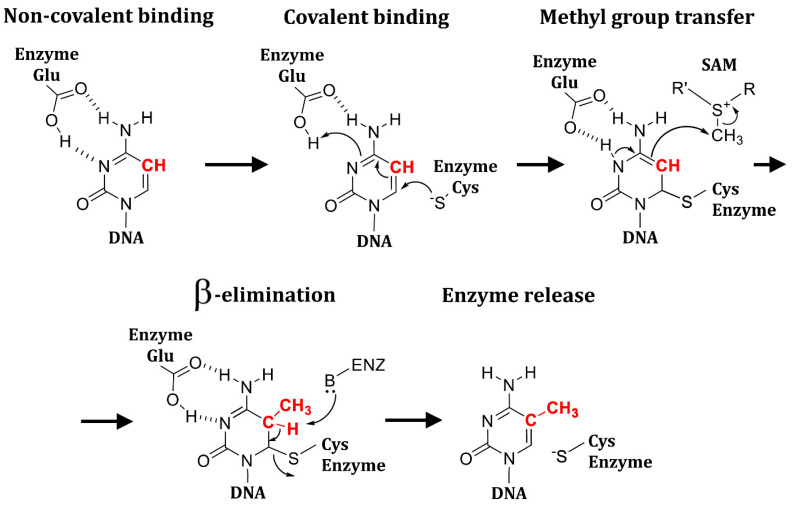
Catalytic mechanism of DNMT1. The C5 atom of the base, which accepts methyl group, is highlighted in red.

The catalytic mechanism of cytosine methylation by DNMTs includes four stages (Figure 7). In the first stage, the binding of the base in the active site occurs, during which H-bonds between the cytosine atoms N3 and N4 and the carboxyl group of the glutamate are formed (Figure 7, top left). Next, protonation of N3 and covalent cysteine binding to the C6 atom take place simultaneously (Figure 7, top middle). In the third step, the methyl group is transferred from SAM to C5 (Figure 7, top right). In the end, the covalent bond between cysteine and C6 is broken by a β-elimination mechanism (Figure 7, bottom left), and the enzyme is released (Figure 7, bottom right) [134].

## 5. Inhibitors of DNA-Methyltransferases

DNA-methyltransferase activity may be disrupted by action on various parts of the enzyme or on different participants of the process. The most attractive target is the enzyme’s active site, which binds two ligands, cytidine and SAM, at once, and the positions of both ligands are well studied.

On the other hand, during the design of the active site inhibitors, special attention should be paid to off-target effects, since neither cytidine nor SAM are substrates unique to DNMTs. This problem can be partially solved by the design of an inhibitor specific to both SAM- and cytidine-binding centers at the same time, which may also increase the effectiveness of the inhibitor.

Most of the inhibitors known today bind to the active site of DNMTs. Nevertheless, the search for allosteric inhibitors is actively underway, and the development of more specific inhibitors interacting with distinctive structural elements outside the active site of DNMT family enzymes is a promising direction of research. Moreover, DNA can also be a target of inhibitors since the intercalation into CpG dinucleotides may lead to disruption of the formation of a catalytically competent complex. In general, all DNMT inhibitors discovered to date can be divided into two main structural groups: nucleoside analogues and compounds of non-nucleoside nature. Non-nucleoside compounds, in turn, are divided into natural and synthetic compounds.

### 5.1. Nucleoside Inhibitors

The first to be designed were nucleoside inhibitors (Table 2), and in particular, 5-aza and decitabine. Their history began with synthesis and research in 1964 [135]. Both were initially considered antimetabolites in AML and showed an antitumor effect [135,136].

The breakthrough came from research on these compounds in small, non-toxic doses. In 1979 and 1980, a research group from the USA showed that the injection of small amounts of 5-aza and decitabine triggers cell differentiation due to DNMT inhibition [137,138]. At the same time, cell reprogramming occurs that includes the activation of the expression of some suppressed genes that leads to the restoration of normal cell functionality.

The mechanism of action of azadeoxynucleosides is based on their phosphorylation and subsequent incorporation into DNA in place of cytidines. Having bound to CpG-dinucleotides, DNMT everts the aza-base as usual, but then, after subsequent covalent binding of the C6-atom to the catalytic cysteine, the following β-elimination step can no longer occur because of the nitrogen atom at the C5-atom’s place [139]. Thus, the enzyme becomes irreversibly bound to DNA, cannot continue its work and is cleaved by proteasomes [139].

Azaribonucleosides are predominantly incorporated into RNA, the consequences of which are not fully studied yet. But azaribonucleosides can be transformed to deoxynucleosides by ribonucleoside reductases, after which they are incorporated into DNA and give the same effect as their deoxy-analogs [140].

In 2004 and 2006, 5-aza (“Vidaza”) and decitabine (“Dacogen”), respectively, became the first nucleoside inhibitors of DNMT, approved for the treatment of myelodysplastic syndrome and chronic leukemia [141]. However, both of these compounds have a number of shortcomings: metabolic instability [142], low bioavailability [143], and also low specificity because of the possibility of incidental incorporation into the DNA of any cell [144]. The metabolic instability and rapid excretion of 5-aza and decitabine are primarily due to the rapid hydrolysis of their bases [142] and susceptibility to deaminases, which also deactivate inhibitors [141]. Moreover, these two compounds have several serious side effects. The combination of these reasons and the development of new drugs have led to their limited use.

Several compounds more stable than 5-aza and decitabine have been proposed (Table 2). 5,6-Dihydro-5-azacytidine had little effect on the rate of methylation processes [145], and its deoxy analogue did not covalently bind to DNMT, exerting an effect only on certain cell lines [146]. Despite reduced toxicity, both of these compounds were withdrawn from clinical trials due to their low efficacy [147]. Deoxyfluorocytidine is also more stable, but is susceptible to deamination and becomes effective only when used in combination with deaminase inhibitors [148]. Zebularine became more well-known [149]. It is a ribonucleoside that also requires a reductase for conversion to a deoxynucleoside. However, the slowdown in action due to extra metabolic steps is offset by the increased effectiveness due to the fact that zebularine itself is a deaminase inhibitor and is not affected by this enzyme [150]. In addition, zebularine does not have extra nitrogen atoms in the base and is therefore more hydrolytically stable. An interesting property of zebularine is that it does not bind covalently to DNMT, since the lack of an amino group prevents the residues of the catalytic center from being positioned in the manner necessary for the reaction [149]. For this reason, its binding to DNMT, although strong, is reversible. Despite its advantages and reduced toxicity, zebularine was not approved for clinical use because its effectiveness was achieved only at high (near-millimolar) doses [151] and mutagenicity in *E. coli* has also been demonstrated [152].

Later, nucleoside compounds were created that are prodrugs of aza-nucleosides. A compound called NPEOC-DAC is a precursor of decitabine and differs from it by the presence of a protective 2-(4-nitrophenyl)ethoxycarbonyl (NPEOC) group [153]. This modification protects aza-cytosine from rapid hydrolysis and is cleaved by carboxylesterase 1, which converts NPEOC-DAC to decitabine. However, due to an additional reaction step, NPEOC-DAC is effective only at high (>10 μM) doses [153].

Compound CP-4200 is a prodrug of 5-aza and differs from it by the presence of an elaidic acid group at the 5′-carbon of deoxyribose [154]. This addition improves the bioavailability of the compound, rendering it independent of membrane transport proteins, but other properties remain unchanged.

The most promising is considered to be the CpG dinucleotide SGI-110, which contains azacitosine [155,156]. The dinucleotide structure protects cytosine from deamination and greatly increases bioavailability [157]. After cleavage of the phosphodiester bond, the released decitabine effectively inhibits DNMT.

### 5.2. Non-Nucleoside Natural Compounds

Any search for compounds capable of altering the efficiency of any enzyme always includes a branch dedicated to reviewing and testing natural substances and known drugs. This is a convenient starting point, as known substances typically do not need to be synthesized from scratch, and if a compound is found to have an effect in vitro, it can be quickly moved into clinical trials.

In the search for DNMT inhibitors, dozens of different known compounds were examined. Among the natural substances studied were gallate epigallocatechin (EGCG), laccaic acid, genistein, psammaplin A, parthenolide, etc. (Table 3). Most of the natural compounds studied, including those named above, were expectedly found to be non-specific: it was shown that even if they do have a demethylating effect, it is indirect and weak [158,159,160,161].

EGCG was shown to noncovalently bind to the catalytic site of DNMT1 with IC50 = 0.47 µM [162]. The authors suggested that EGCG has inhibitory interaction due to the affinity of the gallate moiety to DNMT1. Another compound that has direct inhibitory activity on DNMTs is laccaic acid [163], which was shown to be DNA competitive inhibitor with IC50 = 0.65 µM. Genistein was also associated with DNA demethylation, but different studies showed that it has such a weak inhibitory activity (40% inhibition of DNMT activity at 100 mM) that it cannot be due to its direct action on DNMT [164,165]. As found by Kuck et al. [166], nanaomycin A has a specific affinity for DNMT3B, but another group of scientists later showed that, despite this affinity, this compound does not have an inhibitory effect [167]. Parthenolide has been reported to inhibit DNMT1 with an IC50 = 3.5 µM [161], but its mechanism of action is disputable. Liu et al. suggested that parthenolide directly acts on DNMT1 and covalently binds at its active site, but also it was stated that this compound can block the expression of the DNMT1 gene.

Psammaplin A was suggested to be the first very potent DNMT1 inhibitor with low nanomolar activity (IC50 = 5 nM) [168]. It was also shown to inhibit the histone deacetylase HDAC1 with IC50 = 1 nM, and later studies established that HDAC1 is the only target of psammaplin A [169]. In any case, psammaplin A appeared to be very unstable under physiological conditions and thus was not moved to clinical trials. However, a more stable psammaplin analogue has entered clinical trials as a histone deacetylase inhibitor [170].

### 5.3. Non-Nucleoside Repurposed Compounds

Drug repurposing typically faces the same challenges as natural drugs discovery: low efficacy, low selectivity, and the existence of secondary targets. Among the first drugs tested as DNMT inhibitors were hydralazine [171] and procainamide [172], which are drugs used for arterial hypertension and arrhythmia, respectively (Table 4). The choice of these compounds was not random: a side effect of both drugs was the development of an autoimmune disease, which in turn was accompanied by a disruption of the DNA methylation profile [173]. However, they both showed a low level of DNMT inhibition [174]. Procainamide has been shown to bind to CG-rich regions of DNA with low specificity [175], which is expected to slow down DNMT activity, so the study of this compound continued in an attempt to improve its properties. Unfortunately, only a small improvement to IC50 = 150 μM was achieved [176], so there has been no significant progress in procainamide-based DNMT inhibitor design.

Among the relatively effective known compounds, olsalazine [177,178,179] and liraglutide [180] are also noteworthy (Table 4). Olsalazine is an anti-inflammatory agent derived from salicylic acid. The efficacy and toxicity of this compound are similar to those of decitabine [177,179], but the undoubted advantage of olsalazine is its non-nucleoside nature and low impact on prometastatic genes [177].

Liraglutide is a polypeptide prescribed for type 2 diabetes. The idea to study liraglutide for its epigenetic inhibition of cancer cell proliferation arose after the discovery that another compound, exendin-4, which has the same target, reduced breast cancer cell proliferation [181]. Consequently, a study by Chequin et al. demonstrated that liraglutide is a fairly effective directly acting methyltransferase inhibitor [180]. The computer modeling results predicted a primary effect on DNMT1. Furthermore, the authors report liraglutide’s efficacy in a solid tumor cell model, which has not been achieved in studies of other potential inhibitors.

### 5.4. Designed Non-Nucleoside Inhibitors

#### 5.4.1. Inhibitors Acting on the Active Site

In the mid-2000s, studies began to appear devoted to the search for synthetic compounds capable of inhibiting DNMT. Thus, by means of virtual screening, RG108 was identified [182,183] (Table 5), one of the first synthetic compounds presumably capable of inhibiting DNMT1 by binding at the active site of the enzyme. RG108 showed a weak level of inhibition of DNMT1 (IC50 > 1 mM) [184]; nevertheless, this compound was taken as a basis for the design of more effective inhibitors. In one study, derivatives of RG108 comprising a maleimide group instead of a phthalimide group were studied [184]. It was assumed that this structure of the inhibitor would not disrupt contacts with Arg1308 and Arg1310, while improving interaction with the catalytic Cys1226 residue. The results of this study showed that, indeed, the new compounds possess significantly improved binding capacity, and the best representatives have an IC50 of 10 μM.

Erdmann et al. studied compounds partially similar in structure to RG108 but acting on DNMT3A [185]. Compound 33 (Table 5) demonstrated similar efficacy, with micromolar inhibition constants. It was assumed that the mechanism of binding to DNMT3A is also similar to that of RG108 binding to DNMT1.

Another study attempted to combine RG108 and procainamide into a single molecule [186]. The resulting compounds were expected to have increased effective concentrations due to accumulation at CG-rich sites. Among a series of derivatives, one molecule exhibited good affinity for DNMT1 (IC50 = 5 μM), and another compound exhibited good affinity for DNMT3A and DNMT3L (IC50 = 4 μM).

In the study by Asgatay et al., the authors tested RG108 derivatives, which differed from their precursor in having a more rigid backbone [187]. These compounds were expected to exhibit improved potency due to their form, which is pre-adapted to the active site, and therefore, could bind faster. The best compounds obtained in this study had an IC50 of 20 μM, which is significantly better than RG108, but worse than the results of the studies described above.

Binding at the DNA-binding site and partially at the active site of DNMT1 was predicted using docking for a group of quinoline compounds, including CM-579 [188]. This compound also carries a quinoline group with four substituents. CM-579 strongly binds to DNMT1 (IC50 = 32 nM) and DNMT3A (IC50 = 92 nM), but also has the histone methyltransferase G9a as a secondary target (IC50 = 16 nM). The compound showed good potency in experiments on cells and mice, but its highly uneven distribution throughout the body was noted [189]. Furthermore, recent studies on mice have shown that overdosing on this compound can cause serious side effects, and even death in a number of cases [190].

Newton et al. conducted a search for compounds capable of inhibiting DNMT3B [191]. Compound 33 h, which, according to docking studies, was predicted to bind within the enzyme’s active site, demonstrated the best results (IC50 < 10 μM). According to the model, 33 h completely occupies the active site, interacting with many residues, including one catalytic residue, Arg832.

#### 5.4.2. Inhibitors Acting on the SAM-Binding Site

Some of the first attempts to create inhibitors that bind to the SAM-binding pocket involved altering the cofactor itself. Isakovic at el. conducted an extensive study of S-adenosylhomocysteine analogues, including compounds with altered nitrogenous bases, compounds with altered ribose and compounds with homocysteine analogues [192]. The best results, namely submicromolar IC50 values for DNMT1 and DNMT3B, were demonstrated by compounds in which only the homocysteine was altered, replaced by groups with fewer rotatable bonds (Table 6, the rigid SAG-derivative).

In the study by Halby et al., an attempt was made to create effective inhibitors that bind simultaneously to the SAM-binding pocket and the active site [193]. The idea was to create a molecule containing chemical groups analogous to cytosine and adenine. Quinoline was used as the cytosine analogue, and quinazoline was used instead of adenine. Unlike SGI-1027-like compounds, the authors of this study used six-membered aliphatic heterocycles as the linker of the nitrogenous base analogues. The best results were demonstrated by a compound in which the linker was piperidine, and quinazoline had one highly hydrophobic substituent, biphenyl. The resulting compound 68 (Table 6) had an IC50 of 1.1 μM for DNMT3A and 100 μM for DNMT1.

Another class of compounds that presumably occupy not only the SAM-binding pocket but also part of the active site are furopyrimidinone derivatives [194]. For all of the compounds studied, the authors noted preferential binding to DNMT3A. The best representatives had an IC50 below 1 μM (Table 6, DY-46-2).

In a study by Ceccaldi et al., effective inhibitors were identified among genistein-derived halonitroflavanones by screening [195]. Several molecules demonstrated selective inhibition of specific methyltransferases at the micromolar level (Table 6). Unfortunately, the precise mechanism of inhibition by these compounds has not been elucidated; binding to the SAM-binding pocket was predicted based on docking results.

Affinity to the SAM-binding pocket of DNMT1 was predicted for carbazole-derivatives [196]. In this study, a virtual screening of approximately 200,000 compounds was performed, which resulted in the identification of one compound (Table 6, DC_517) with an IC50 of 1.7 μM. Selectivity relative to other methyltransferases was demonstrated, but the overall selectivity remains questionable.

#### 5.4.3. Allosteric Inhibitors

The search for allosteric inhibitors of any enzyme is usually difficult since it is more difficult to determine the exact mechanisms of interactions of molecules outside the active center and such interactions are often weaker than interactions with the substrate.

Nevertheless, one group of scientists managed to identify allosteric inhibitors of DNMT3A [197] (Table 7). In this study, a library of compounds with efficacy against various tropical diseases was screened. The screening yielded one representative from each of two similar chemical families of compounds that did not compete with either DNA or the cofactor. The selected compounds were elongated molecules containing pyrazolone and pyridazine moieties. Both compounds were shown to have micromolar IC50 values for DNMT3A but were significantly less effective at inhibiting DNMT1, with the pyridazine variant being more selective.

#### 5.4.4. Inhibitors Acting on DNA

In a study by Hossain et al., compounds that reduce DNA methyltransferase activity were unexpectedly identified using cell culture screening [198]. These compounds, however, bind to DNA rather than the enzyme itself. The compounds contained an acridine group (Table 7). It is known from the literature that such compounds can intercalate into DNA [199], so what was even more surprising was that the compounds were selective enough to activate important methylated genes in various cancer cell lines. Moreover, the effective concentrations of the new compounds were in the micromolar range.

A compound called SGI-1027 was the first quinoline compound to inhibit methyltransferases through binding to DNA. The precise mechanism of SGI-1027 inhibition was later elucidated by Gros et al., and it was shown that SGI-1027 competes with SAM, but not with DNA, for binding to methyltransferases [200]. Several analogues of SGI-1027 were also investigated in the same study. Compound 31 (Table 8, SGI-1027 derivative No. 31), in which the carbonyl and amino groups at the backbone were swapped and the substituents were removed from one aromatic ring, demonstrated reduced inhibitory potency. Meanwhile, compound 5 (Table 8, SGI-1027 derivative No. 5), in which the benzene groups at the backbone were substituted in the meta rather than para positions, exhibited slightly higher inhibitory potency and also bound more strongly to DNA. An interesting result was that an SGI-1027 analogue of the same form, but lacking the quinoline group, does not bind to DNA and is an order of magnitude weaker inhibitor than SGI-1027.

The study of quinoline compounds was continued by Zhou et al. [201]. In this work, several analogues of SGI-1027 were studied, their ability to inhibit various DNA-binding enzymes was investigated and several crystal structures in complex with DNA and the adenine methyltransferase CamA were obtained. As revealed from crystal structures, SGI-1027 analogues were not specific to CpG dinucleotides and preferred to bind between G-C and A-T pairs, intercalating into the DNA helix with their quinoline group, which is why they exhibit non-specific inhibition of various DNA-binding enzymes, including the bacterial adenine methyltransferases Dam and CcrM, DNA polymerase θ and HIV reverse transcriptase. Several compounds exhibited an inhibitory effect at the level of several μM against DNMTs. Compound 5, obtained in the work described earlier [200], can be considered the best, since it has a submicromolar IC50 in relation to DNMTs, while having an IC50 for other enzymes of at least 10 μM.

Compounds of a different chemical class inhibiting DNMT1 by intercalating into DNA were discovered relatively recently [202]. The new compounds were based on a dicyanopyridine group with three additional substituents. The first compound identified, named GSK3484862 (Table 8), exhibited strong inhibitory activity against DNMT1 (IC50 = 230 nM) and high selectivity for it: the inhibition of other methyltransferases did not occur even at 50 μM inhibitor concentrations. Subsequent structure optimization yielded the compound GSK3685032, whose inhibitory activity was an order of magnitude higher (IC50 = 36 nM).

The improved compound GSK3685032 has already been tested in cell and murine models [202]. Cell line studies showed a significant increase in the expression of various methylated genes, with the greatest increase in expression observed for genes with, initially, the most methylated promoters. Subcutaneous administration of the drug to murine xenograft tumor models demonstrated a statistically significant slowdown in tumor growth, while doses sufficient to inhibit tumor growth were nontoxic, unlike similar doses of decitabine, used as a control. In the same study, it was also demonstrated that all of the selected compounds bind specifically to the DNMT1-DNA complex and do not bind to the enzyme or DNA separately. Furthermore, the compounds exhibited high selectivity for CpG pairs methylated in only one DNA strand.

**Figure 8:**
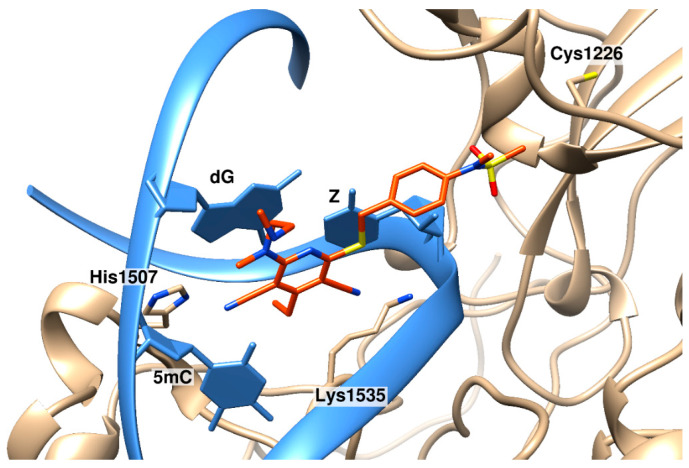
The complex of DNMT1 (beige) with DNA (blue) containing zebularine (Z) and intercalated GSK3830052 (orange) (PDB ID 6X9J [203). The dicyanopyridine moiety of the inhibitor intercalates between two G-C pairs. One cyano group is next to His1507 on the major groove side, the other is next to Lys1535. The sulfo group is located near the active site.

Later, the same group of authors made a second attempt to elucidate the reasons for such remarkable selectivity of the obtained compounds [203]. For this purpose, a few more dicyanopyridine compounds with altered substituents were synthesized. Unfortunately, the new compounds did not exhibit improved properties, but for these compounds, as well as for the previous compounds, the authors obtained crystal structures of the ternary complex with DNMT1 and DNA containing zebularine to increase the complex’s stability. Based on the structural data, important conclusions were made: firstly, intercalation into DNA occurs from the minor groove side by the dicyanopyridine group, and strong binding requires the His1507 residue, located near the guanine opposite the target cytosine, which interacts with one of the cyano groups (Figure 8). An important feature is that His1507 is the residue located in a large variant of TRD that is present only in DNMT1. Substitution of this residue with alanine and tyrosine led to a decrease in the inhibitory activity of the compounds. These findings may well explain both the necessity of the DNMT1-DNA complex for inhibitor binding and its selectivity for CpG pairs and DNMT1. Secondly, bulkier pyridine substituents interfere with the protein loop 1226–1236, which contains active site residues, including Cys1226, presumably preventing the achievement of a catalytically competent state. Furthermore, it was discovered that suitable pockets exist on the protein surface for pyridine substituents, which could be exploited to improve inhibitor binding.

## 6. Conclusions and Perspectives

DNA methyltransferases serve as central enzymes in the epigenetic control of gene activity, dynamically shaping methylation patterns across embryonic and mature cells. DNMT misfunctions can trigger severe disorders and cancers by disrupting gene methylation. This review covers the biological roles of DNMTs and their structural features, along with the evolution of DNMT inhibitor development. A wide range of known DNA methyltransferase inhibitors belonging to completely different structural families currently exist, differing in both their mechanisms of action and selectivity. Nucleoside analogues have gained widespread popularity due to their well-understood mechanism of action and reasonable efficacy. However, their high toxicity and other side effects prevented them from becoming the definitive anticancer drugs or methyltransferase activity modulators. Despite significant efforts to find more effective inhibitors, new compounds that outperform nucleoside analogues in all necessary criteria remain elusive. A breakthrough in this area was achieved by the discovery of cyanopyridine compounds that are not only highly effective but also exhibit a novel, unusual mechanism of action worthy of special attention. Nevertheless, there is still a lack of information on the structures of enzyme–inhibitor complexes and the chemical mechanisms of action of many inhibitors, which slows the development of new, potent compounds and also does not allow this review to examine the relationship between the chemical structure of compounds and the specificity and efficacy of action and to conduct a full comparative characterization of inhibitors. Thus, further research on inhibitors acting directly on DNMTs and, especially, selective against DNMT3A/B would be very useful. This review summarizes valuable insights into approaches for inhibiting methyltransferases and data on the latest developments in new, effective and selective compounds.

## Figures and Tables

**Table 1:** Summary table of mammalian DNMTs in normal and pathological states. ADCA-DN—autosomal dominant cerebellar ataxia, deafness and narcolepsy; HSAN1E—hereditary sensory and autonomic neuropathy, type 1E; AML—acute myeloid leukemia; T-ALL—T-cell acute lymphoblastic leukemia; ICF—immunodeficiency–centromeric instability–facial anomalies.

Enzyme	Canonical Activity/Substrate	Main Physiological Functions	Major Pathological Associations (Examples)
DNMT1	Maintenance DNA methyltransferase; mainly CpG methylation [7].	Preserves DNA methylation patterns during DNA replication [8]; essential for embryonic development, neuronal viability, chromatin organization, and cell-cycle regulation [8,17,18].	ADCA-DN and HSAN1E development [18,21,24,26]; psychiatric and addictive-like phenotypes in humans [30,31,34,35,36,37] and experimental models [32,33]; cardiac fibrosis and heart failure [38,39,40,41]; disuse osteoporosis; Graves’ disease; age-related hypomethylation and genomic instability [42]; overexpression in breast cancer and melanoma [48,49,50].
DNMT3A	De novo DNA methyltransferase; CpG and some non-CpG methylation [5,6,7,8,9].	Establishes DNA methylation during development [5,6,7,8,9]; involved in methylation of *Xist* and imprinted genes in germ cells [51]; contributes to neuronal plasticity and long-term memory in rodent models [52].	Tatton–Brown–Rahman syndrome [53,54]; microcephalic dwarfism [57]; AML, myelodysplastic syndrome, and T-ALL [53,55,56,60,61]; addictive-like behavior in rodent models [58,59]; low-grade glioma and breast cancer [48,64].
DNMT3B	De novo DNA methyltransferase; mainly CpG methylation [5,6,7,8,9].	Establishes methylation patterns during embryogenesis [69]; cooperates with DNMT3A in de novo methylation [8].	ICF syndrome [8,70,71]; increased breast cancer risk [72,73,74]; pro-tumorigenic role in melanoma and colon cancer [75,76]; regulation of macrophage polarization in obesity in mice models [77]; pulmonary fibrosis in mice models [78]; allergic rhinitis models [79].
DNMT2	Non-canonical RNA methyltransferase; methylates C38 in tRNA(Asp) [13,80,81,82,83].	Supports tRNA stability and participates in antiviral responses [81,82,83].	Upregulated in low-grade glioma [64]; knockout in glioblastoma cells associated with altered drug sensitivity [84]; broader role in human disease remains unclear.
DNMT3L	Catalytically inactive DNMT-like protein [7].	Lacks intrinsic methyltransferase activity; acts as a regulatory cofactor for DNMT3A/3B [85,86,87,88].	No direct catalytic disease mechanism established; may indirectly modulate methylation-related disorders through altered regulation of DNMT3A/3B.

**Table 2:** Nucleoside analog DNMT inhibitors.

5-aza	Decitabine	5,6-dihydro-5-azacytidine	Deoxyfluorocytidine
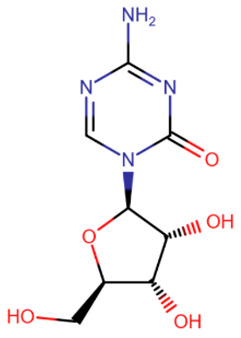	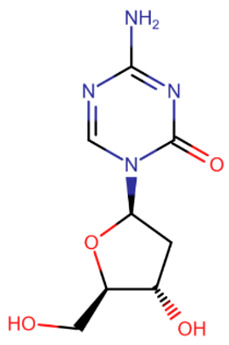	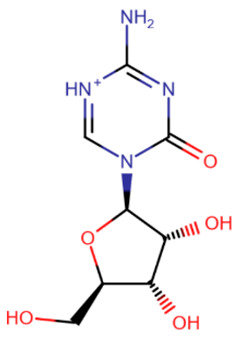	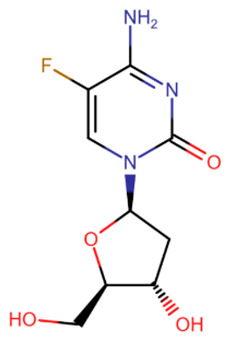
Zebularine	NPEOC-DAC	CP-4200	SGI-110
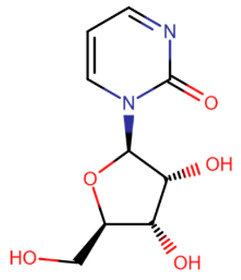	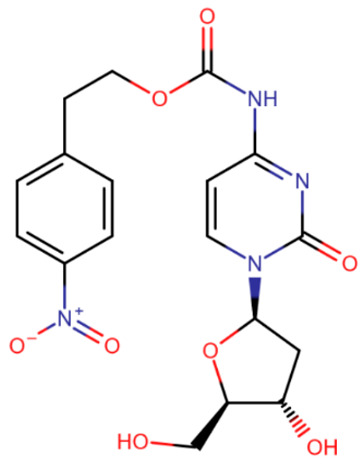	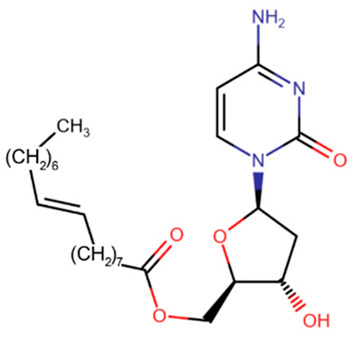	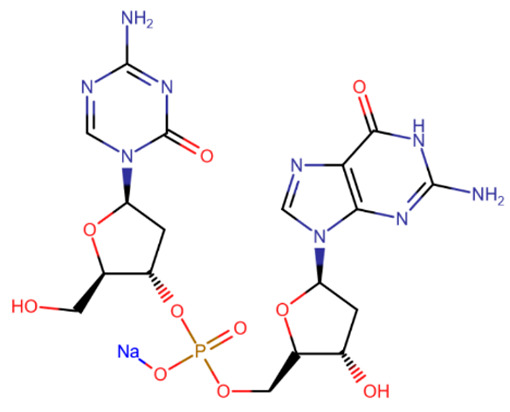

**Table 3:** Natural compounds studied for inhibition of DNMTs.

EGCG	Laccaic acid	Genistein
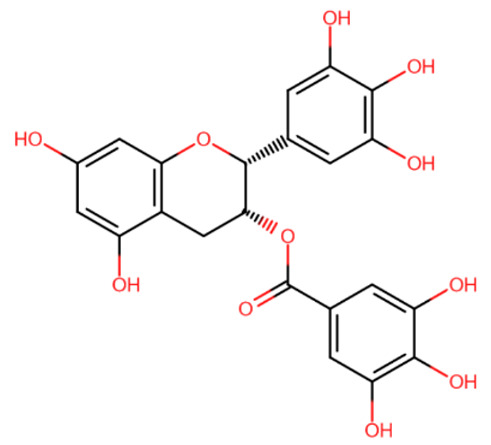	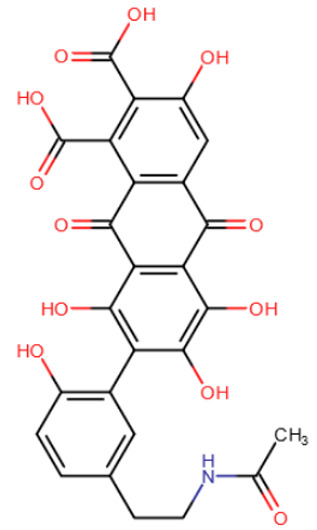	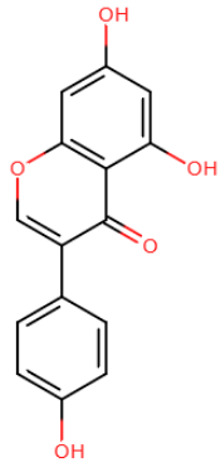
Psammaplin A	Parthenolide	
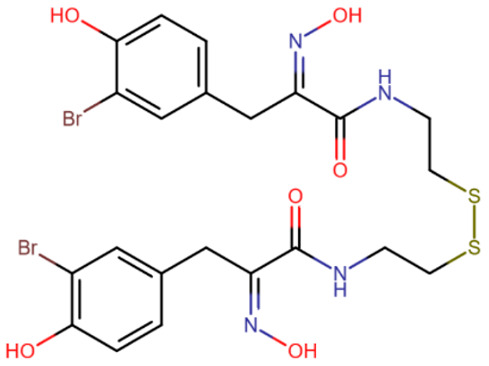	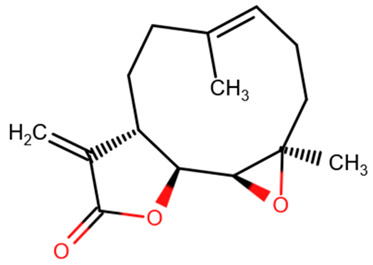	

**Table 4:** Known drugs that have been tested for DNMT inhibition.

Parthenolide	Olsalazin	Nanaomycin A	Hydralazine	Procainamide
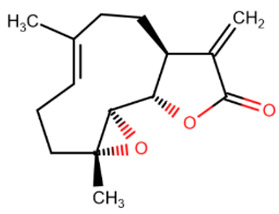	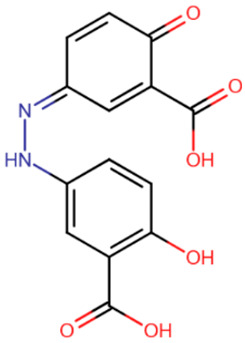	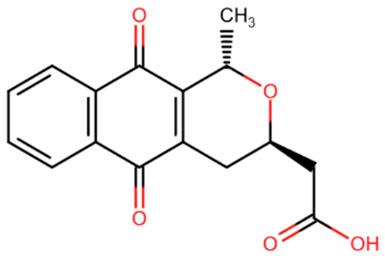	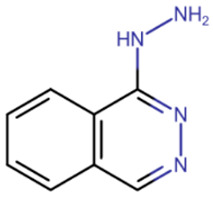	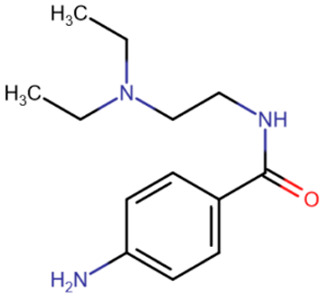

**Table 5:** Inhibitors acting on the active site of DNMTs.

RG108	Maleimide RG108-derivative	Compound 33	Rigid RG108-derivative
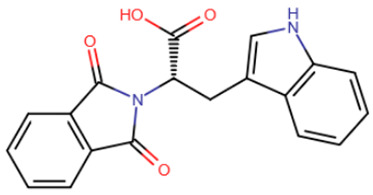	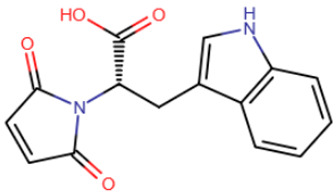	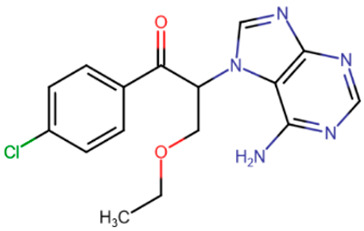	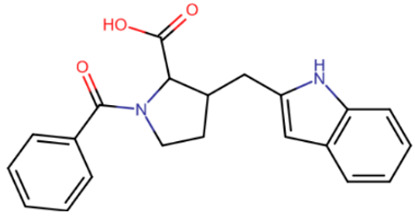
RG108-procainamide fusion	SGI-1027	CM-579	
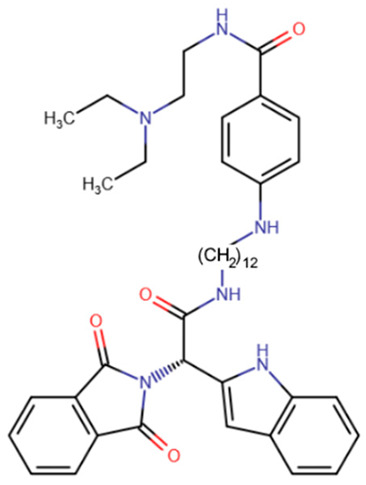	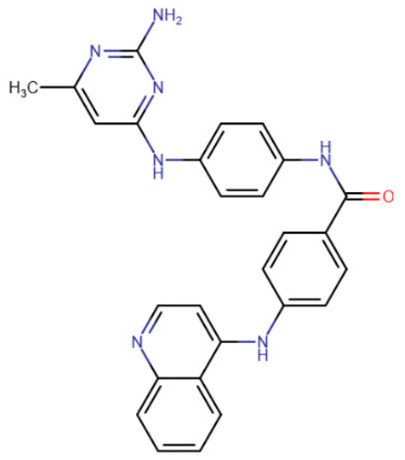	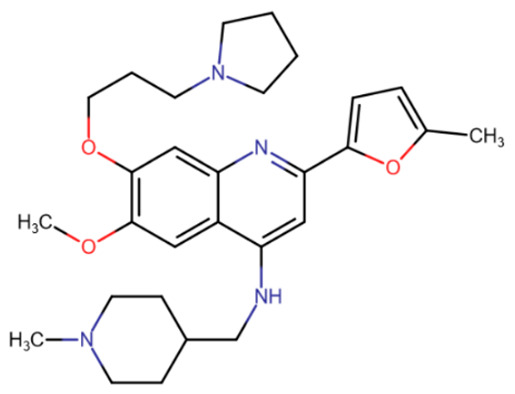	
33 h			
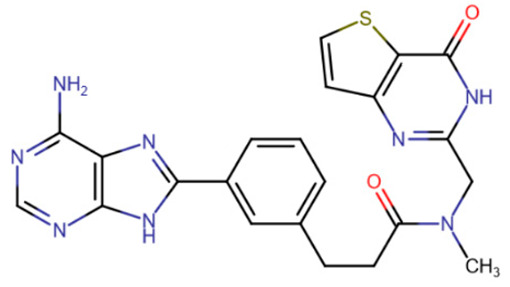			

**Table 6:** Inhibitors interacting with the SAM-binding site.

Rigid SAH-derivative	DC_517	Halonitroflavanone derivative
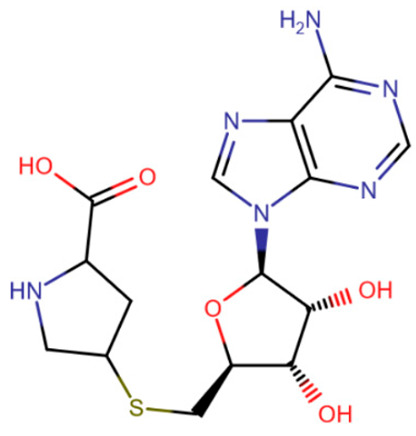	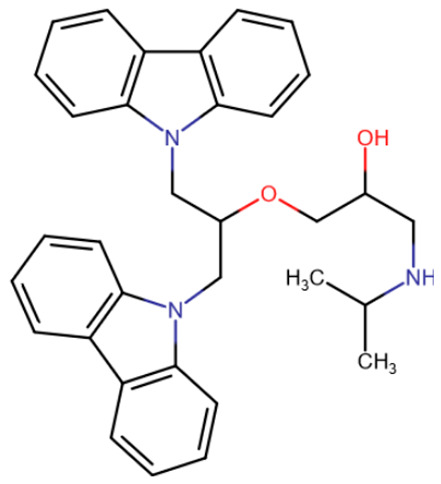	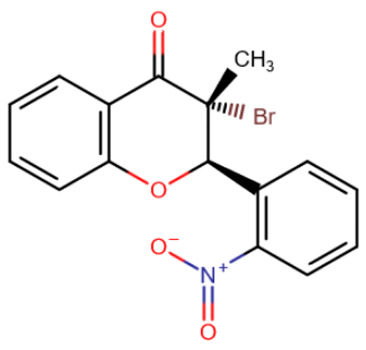
DY-46-2	Bisubstrate inhibitor No. 68	
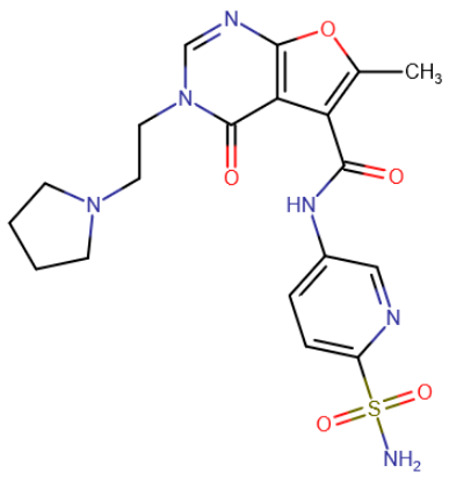	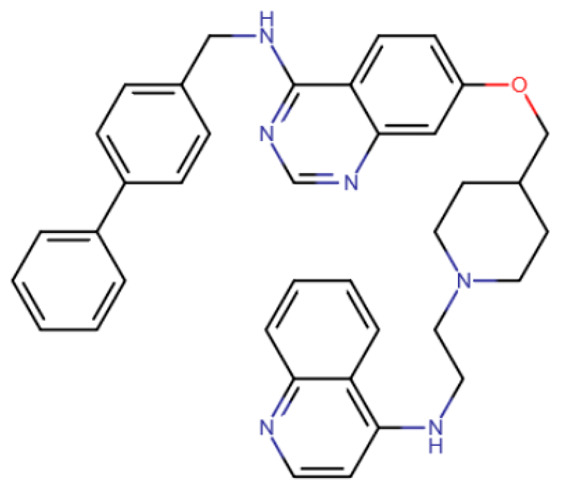	

**Table 7:** Allosteric inhibitors of DNMTs.

Pyrazolone derivative	Pyridazine derivative
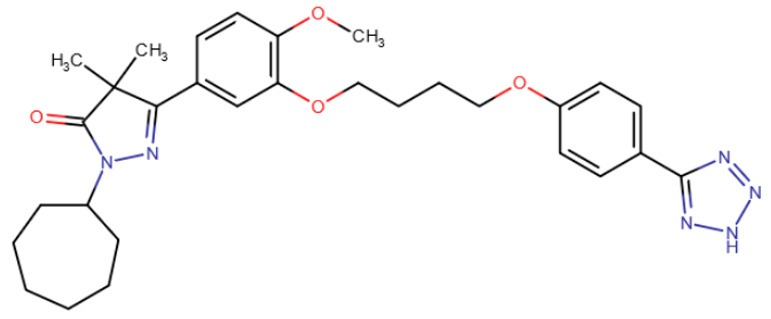	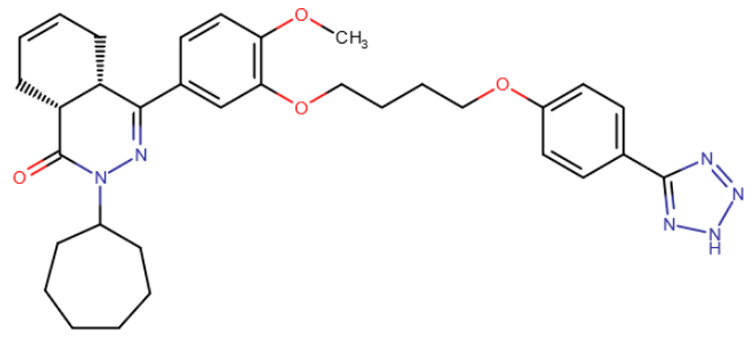

**Table 8:** DNMT inhibitors intercalating into DNA.

Acridine derivative	SGI-1027 derivative No. 5	SGI-1027 derivative No. 31	GSK3484862
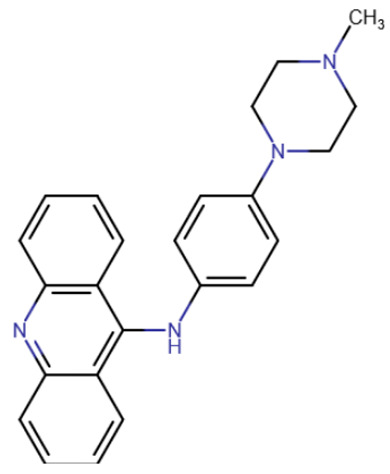	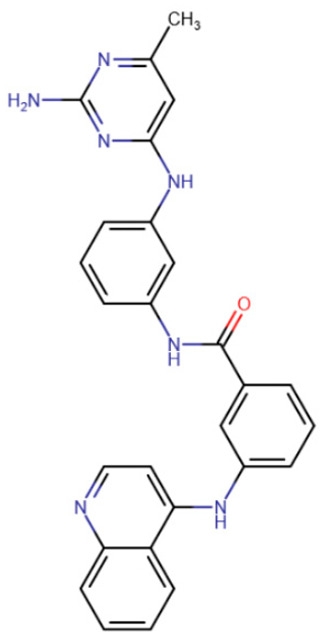	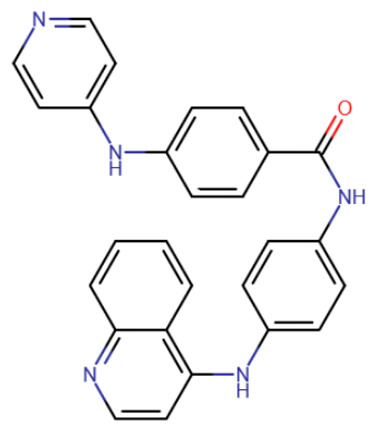	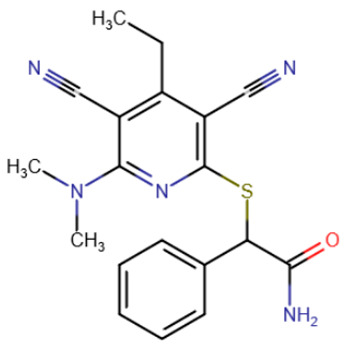
			GSK3685032
			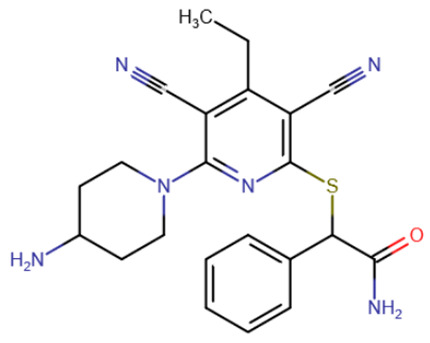

## Data Availability

All data supporting the findings of this study are available within the paper.

## Abbreviations

The following abbreviations are used in this manuscript:

DNMT	DNA methyltransferase
SAM	S-adenosylmethionine
DMAP1	DNA methyltransferase-associated protein-1 binding domain
PCNA	proliferating cell nuclear antigen
PBD	PCNA binding domain
NLS	nuclear localization signal
RFTS	replication foci-targeting sequence domain
BAH	bromo-adjacent-homology domain
PWWP	Pro-Trp-Trp-Pro domain
ADD	ATRX-DNMT3-DNMT3L domain
CXXC	cysteine-rich subdomain
SNP	single nucleotide polymorphism
TRD	target recognition domain
